# Autophagy in spinal ligament fibroblasts: evidence and possible implications for ossification of the posterior longitudinal ligament

**DOI:** 10.1186/s13018-020-02017-6

**Published:** 2020-10-22

**Authors:** Yuehua Yang, Zunwen Lin, Jiangwei Chen, Sheng Ding, Weiwei Mao, Sheng Shi, Biru Liang

**Affiliations:** 1 Department of Orthopaedics, The Fifth Affiliated Hospital, Southern Medical University, No. 566 Congcheng Avenue, Conghua District, Guangzhou, 510900 People’s Republic of China; 2 Department of Orthopedic Surgery, The First Affiliated Hospital, Nanchang University, No. 17, Yongwaizheng Street, Nanchang, 330006 Jiangxi People’s Republic of China; 3 Department of Stomatology, Xinhua Hospital Affiliated to Shanghai Jiao Tong University School of Medicine, 1665, Kongjiang Road, Shanghai, 200092 People’s Republic of China; 4 Department of Pediatric Neurosurgery, Xinhua Hospital Affiliated to Shanghai Jiao Tong University School of Medicine, 1665, Kongjiang Road, Shanghai, 200092 People’s Republic of China; 5 Department of Orthopedics, Shanghai Tenth People’s Hospital, School of Medicine, Tongji University, 301 Middle Yanchang Road, Shanghai, 200072 People’s Republic of China

**Keywords:** Autophagy, Ossification of the posterior longitudinal ligament, Fibroblast, Osteogenic differentiation

## Abstract

**Background:**

The molecular mechanisms of ossification of the posterior longitudinal ligament (OPLL) remain to be elucidated. The aim of the present study was to investigate the autophagy of spinal ligament fibroblasts derived from patients with OPLL and to examine whether autophagy-associated gene expression was correlated with the expression of osteogenic differentiation genes.

**Methods:**

Expression of autophagy-associated genes was detected in 37 samples from 21 OPLL patients and 16 non-OPLL patients. The correlation of autophagy-associated gene expression and the expression of osteogenic differentiation genes was analyzed by Pearson’s correlation. The expression of autophagy-associated genes of ligament fibroblasts was assessed by reverse transcription-quantitative polymerase chain reaction (RT-qPCR), western blotting, and immunofluorescence. The incidence of autophagy was assessed by flow cytometry. After knockdown using small interfering RNA targeting Beclin1, the expression of osteogenic differentiation genes were compared in spinal ligament fibroblasts.

**Results:**

In clinical specimens, mRNA expression levels of microtubule-associated protein 1 light chain 3 and Beclin1 were higher in the OPLL group compared with the non-OPLL group. Pearson correlation analysis demonstrated that Beclin1 expression was positively correlated with expression of osteocalcin (OCN) (*r* = 0.8233, *P* < 0.001), alkaline phosphatase, biomineralization associated (ALP) (*r* = 0.7821, *P* < 0.001), and collagen type 1 (COL 1) (*r* = 0.6078, *P* = 0.001). Consistently, the upregulation of autophagy-associated genes in ligament fibroblasts from patients with OPLL were further confirmed by western blotting and immunofluorescence. The incidence of autophagy was also increased in ligament fibroblasts from patients with OPLL. Furthermore, knockdown of Beclin1 led to a decrease in the expression of OCN, ALP, and COL 1 by 63.2% (*P* < 0.01), 52% (*P* < 0.01), and 53.2% (*P* < 0.01) in ligament fibroblasts from patients with OPLL, respectively.

**Conclusions:**

Beclin1-mediated autophagy was involved in the osteogenic differentiation of ligament fibroblasts and promoted the development of OPLL.

## Introduction

Ossification of the posterior longitudinal ligament (OPLL) is characterized by heterotopic ossification in the spine ligament [1, 2]. At present, several genetic and non-genetic factors are involved in the pathological progress of OPLL. Aberrant levels of fibroblast growth factor-23 [3], leptin [4], and dickkopf-1 and sclerostin [5], as well as mechanical stress signaling, have been identified as main contributing factors in the development of OPLL [6–8].

Autophagy, a macromolecular degradation process, plays a pivotal role in the maintenance of cell differentiation [9]. Autophagy of osteocytes was first confirmed by Zahm et al. [10], and subsequent studies had reported that autophagic receptor and Unc-51 like autophagy activating kinase 1 (ULK1) had an important effect on the activity and differentiation of osteoblasts [11–13]. Consistently, another study demonstrated that osteoblastic differentiation was dependent on Beclin1 dependent autophagy [14]. Microtubule-associated protein 1 light chain 3 (LC3), a lipidated protein specifically associated with the membranes of the autophagosomes, was also correlated with osteoblastic differentiation [15]. Furthermore, our previous results revealed that the level of osteocyte autophagy was associated with bone loss in ovariectomized rats and aged rats [15–17]. Hitherto, it is generally accepted that autophagy plays an essential role in the osteogenic differentiation of mesenchymal stem cells [14, 18]. However, autophagy of spinal ligament fibroblast has not yet been investigated and the underlying role of autophagy in OPLL processes remains unknown.

Based on the aforementioned data, it is speculated that autophagy is associated with the differentiation of osteoblasts. We hypothesized that the ossification of ligament fibroblasts may be attributed to abnormal autophagy. To confirm this hypothesis, a clinical study was carried out on 37 subjects (21 cases of OPLL patients and 16 cases of non-OPLL patients). The main objectives of this research are as follows: (1) to compare the autophagy level of spinal ligament fibroblasts between OPLL and non-OPLL patients and (2) to investigate the association between autophagy level and the expression of osteogenic differentiation genes.

## Materials and methods

### Characterization of patients

The present study was approved by The Fifth Affiliated Hospital, Southern Medical University, Shanghai Tenth People’s Hospital, Tongji University, and Xinhua Hospital Affiliated to Shanghai Jiao Tong University School of Medicine (approval no. XHEC-D-2015-112). The primary endpoint is LC3 mRNA relative expression level between OPLL and non-OPLL patients. According to our previous preliminary test (*n* = 5 for each group), the relative expression of LC3 mRNA in the control group was 0.28 ± 0.13 (mean ± standard deviation). The relative mean expression in OPLL group was 0.51 ± 0.21. Based on a two-sided 5% significance level and a power of 90%, sample size was calculated using PASS 11 statistical software (version 11.0.8 for Windows 7). A sample size of 12 subjects in each group was necessary to detect a difference expression of LC3 mRNA between the OPLL and control groups. From December 2015 to December 2018, X-ray, CT images, and MRI of the cervical spine were evaluated preoperatively in each patient. According to inclusion and exclusion criteria for patients referenced previous methods [19, 20], actually, in this study, 21 cases of OPLL patients and 16 cases of non-OPLL patients were enrolled. All patients underwent anterior cervical decompression surgery and all surgical procedures were performed by the same five surgeons.

All patients provided written informed consent and spinal ligament tissues were obtained during surgery using intraoperative aseptic techniques. The OPLL patients with a radiographic diagnosis of OPLL involving the cervical spine, as well as symptoms, such as neck pain and numbness in the extremities. OPLL patients included 10 males and 11 females, with a mean age of 48 ± 5.9 years. Ossified lesions were distributed from C1 to C7, including 4 cases of continuous type, 2 cases of mixed type, 1 case of segmental type, and 14 case of local type. By contrast, 16 non-OPLL patients with cervical trauma spinal fracture were enrolled, who did not have OPLL, cervical spondylosis or stenosis. In the non-OPLL group, the patients included 9 males and 7 females, with a mean age of 42.1 ± 13.1 years.

To investigate this hypothesis, autophagy was assessed by reverse transcription-quantitative (RT-q)PCR, western blotting, and immunofluorescence in ligament clinical specimens and ligament fibroblasts. Subsequently, Pearson’s correlation was used to compare the expression of osteogenic-specific genes with autophagy. Besides, small interfering (si)RNA was used to knockdown the expression of Beclin1 in spinal ligament fibroblasts, following which the expression levels of osteocalcin (OCN), alkaline phosphatase, biomineralization associated (ALP), and collagen type 1 (COL 1) were compared in OPLL ligament fibroblasts and non-OPLL ligament fibroblasts.

### Reagents and antibodies

Monodansylcadaverine (MDC) was purchased from Sigma-Aldrich, Merck KGaA. Polyclonal antibodies against Beclin1 and LC3 were obtained from Cell Signaling Technologies, Inc. β-actin, vimentin, and keratin antibodies were obtained from Abcam.

### Specimen processing and cell isolation

During the anterior cervical decompression surgery, 37 posterior longitudinal ligament specimens were obtained. To avoid contamination with osteoblasts or osteocytes, the ligaments were extracted carefully, cut up into 1-mm^2^ pieces and washed with PBS several times. Subsequently, the specimens were divided into two parts. One was stored in liquid nitrogen for reverse transcription-quantitative polymerase chain reaction (RT-qPCR) analysis. The remaining were washed with normal saline, plated in 35-mm culture dishes, and maintained in Dulbecco’s modified Eagle’s media supplemented with 10% fetal bovine serum (Gibco, USA). All assays were carried out on the fifth passage cell cultures.

### Measurement of cell viability

Cell viability was examined using 2-(2-methoxy-4-nitrophenyl)-3-(4-nitrophenyl)-5-(2,4-disulfo-phenyl)-2H-tetrazolium (Beyotime Institute of Biotechnology), according to the manufacturer’s protocols. Briefly, the ligament fibroblasts were plated in 96-well culture plates and cultured in the osteogenic differentiating medium for 1, 2, 3, 4, 5, 6, 7, or 8 days. Cell viability assays were performed and the absorbance of optical densities were measured at each time point and detected by a microplate spectrophotometer at 450 nm.

### RT-qPCR

Total RNA was obtained from the posterior longitudinal ligament specimens or the ligament fibroblasts, according to our previous study design. Expression of Beclin1, LC3, ULK1, COL 1, OCN, and ALP were examined by RT-qPCR, according to our previous method [16]. For PCR amplification, specific oligonucleotide primers of rat sequences were designed on the basis of sequences in GenBank (Table 1).

**Table 1:** Primer sequences of Beclin1, LC3, ULK1, OCN, ALP, COL I, and β-actin

Index	Primer sequences
Beclin1	
Forward	5′-CAGGAACTCACAGCTCCATT-3′
Reverse	5′-CATCAGATGCCTCCCCAATC-3′
LC3	
Forward	5′-AGCCACCTGCCACTCCTGAC-3′
Reverse	5′-ACCTTCCCTGCTGCCCTCAC-3′
ULK1	
Forward	5′-GAGTCGGAGTCGGAGTCGGATC-3′
Reverse	5′-CGAACTTGCCCACGGTCTCTG-3′
OCN	
Forward	5′-AGGGCAGCGAGGTAGTGA-3′
Reverse	5′-CCTGAAAGCCGATGTGGT-3′
ALP	
Forward	5′-GTGGACTATGCTCACAACAA-3′
Reverse	5′-GGAGAAATACGTTCGCTAGA-3′
COL I	
Forward	5′-CGAAGACATCCCACCAATC-3′
Reverse	5′-ATCACGTCATCGCACAACA-3′
β-actin	
Forward	5′-CTCCATCCTGGCCTCGCTGT-3′
Reverse	5′-GCTGTCACCTTCACCGTTCC-3′

### Western blotting

The protein expression of Beclin1, LC3-II/I, β-actin, and Glyceraldehyde-3-phosphate dehydrogenase (GAPDH) was detected by western blotting, according to our previous paper [15]. Briefly, total protein was extracted using a western blot kit (Beyotime Institute of Biotechnology). Each sample (50 μg/well) was separated on a 10% sodium dodecyl sulfate-polyacrylamide gel for Beclin1 protein and 12% sodium dodecyl sulfate-polyacrylamide gel for LC3 protein. Next, separated proteins were transferred to polyvinylidene difluoride membranes, followed by blocking with 5% non-fat milk for 1 h. Next, the polyvinylidene difluoride membranes were incubated with anti-β-actin (Cat. #4970; CST, USA) (dilution 1:1000), GAPDH (Cat. #2118; CST, USA) (dilution 1:1000), anti-Beclin1(Cat. #3495; CST, USA) (dilution 1:500), or anti-LC3 antibodies (Cat. #83506; CST, USA) (dilution 1:200) and detected using the ECL detection kit (Beyotime Institute of Biotechnology). The blots were quantified by densitometry using Image Lab version 2.1 software (Bio-Rad Laboratories, Inc.).

### Knockdown of Beclin1 in ligament fibroblasts

siRNAs specifically targeting Beclin1 were constructed and designed by superbiotek (Shanghai, China). To target Beclin1, the following sequences were used: forward, 5′-GAGCGAUGGUAGUUCUGGAGG-3′ and reverse, 3′-UCCAGAACUACCAUCGCUCUG-5′. A missense siRNA vector (lack of complementary sequences) served as a non-silencing control (siControl). All transfections were carried out using Lipofectamine® 2000 (Invitrogen; Thermo Fisher Scientific, Inc.) according to the manufacturer’s instructions.

### Levels of autophagy and apoptosis quantified by flow cytometry

The rates of apoptosis were analyzed by flow cytometry in the present study, according to our previous method [16]. The rates of autophagy were analyzed by flow cytometry according to the method of Bursch et al. [21] and Shen et al. [22]. Briefly, the cells were incubated with 0.05 mM MDC (Cat. #30432; Sigma-Aldrich, USA) in PBS at 37 °C for 10 min and then the intracellular MDC was measured by flow cytometry within 30 min. The autophagy incidence was determined as the percent of MDC positive cells automatically analyzed using FlowJo software (Tree Star, San Carlos, CA, USA) and the unstained cells were used as control.

### Immunofluorescence for vimentin and keratin

The ligament fibroblasts were seeded on 6-well plates with sterile glass cover slip, fixed with 4% paraformaldehyde and permeabilized in 0.1% Triton X-100 for 20 min. Subsequently, the ligament fibroblasts were incubated with primary anti-vimentin (Cat. #5741; CST, USA) (dilution 1:200) or anti-keratin antibodies (Cat. #13063; CST, USA) (dilution 1:200) for 2 h. This was followed by incubation with an Alexa Fluor® 488-conjugated secondary antibody (Cat. #4408; CST, USA) (dilution 1:200) for 1 h, and images were taken with a fluorescence microscope (Olympus Corporation).

### Immunofluorescence for LC3 and Beclin1 protein

The ligament fibroblasts were seeded on 6-well plates with sterile glass cover slips and transfection of LC3 protein fused with green fluorescent protein plasmid (GFP-LC3) was carried out using Lipofectamine 2000 (Invitrogen; Thermo Fisher Scientific, Inc.), as previously described [23]. Subsequently, cells were incubated for 72 h and then fixed with 4% paraformaldehyde for 15 min, followed by incubation with anti-Beclin1 (Cat. #3495; CST, USA) (dilution 1:200) in 5% bovine serum albumin (Cat. # ST023; Beyotime, China) overnight at 4 °C. Subsequently, the ligament fibroblasts were incubated with Alexa Fluor® 594-conjugated secondary antibody (Cat. #8889; CST, USA) (1:100) and then observed by fluorescence microscopy (Olympus Corporation).

### Statistical analysis

All data were presented as the mean ± SD. Differences in mRNA expression among the OPLL and non-OPLL groups were analyzed using a *t* test, with SPSS 13 (SPSS, Inc.). Multiple comparisons of data among the groups were determined by the one-way ANOVA followed by Dunnett’s test. Pearson correlation coefficients were used to analyze the correlation between parameters of autophagy level and osteogenic makers. *P* < 0.05 was considered to indicate a statistically significant difference.

## Results

### High expression of Beclin1 and LC3 mRNA in OPLL tissues

**Fig. 1:**
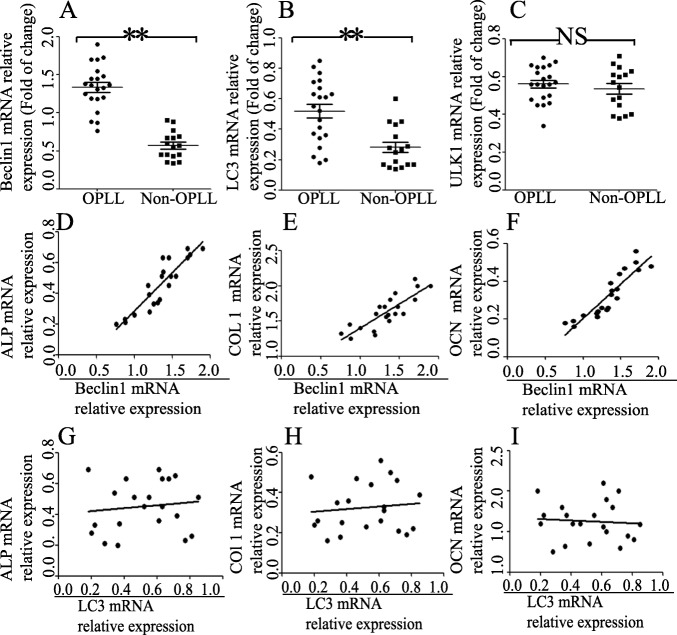
Expression of Beclin1 and LC3 mRNA in OPLL tissues and correlation between autophagy level and osteogenic differentiation markers. Reverse transcription-quantitative PCR assay to measure the mRNA expression of Beclin1 (**a**), LC3 (**b**), and ULK1 (**c**) in spinal ligament tissues from patients with OPLL and non-OPLL patients (*n* = 21 for OPLL group; *n* = 16 for OPLL group). The relative gene expression was normalized against β-actin. *P < 0.05, **P < 0.01. Pearson’s correlation was used to analyze the correlation between Beclin1 level and osteogenic biomarkers ALP (**d**), COL 1 (**e**), and OCN (**f**) in spinal ligament tissues from patients with OPLL and non-OPLL patients (*n* = 21 for OPLL group; *n* = 16 for OPLL group). Correlation between LC3 level and ALP (**g**), COL 1 (**h**), and OCN (**i**) spinal ligament tissues from patients with OPLL and non-OPLL patients (*n* = 21 for OPLL group; *n* = 16 for OPLL group). LC3, microtubule-associated protein 1 light chain 3; OPLL, ossification of the posterior longitudinal ligament; ULK1, Unc-51 like autophagy activating kinase 1; ALP, alkaline phosphatase, biomineralization associated; COL 1, collagen type 1; OCN, osteocalcin

In this part of the study, expression levels of different autophagy markers in the spinal ligament tissues from OPLL and non-OPLL patients were investigated. The present results reported that the expression of Beclin1 was significantly higher in the OPLL group compared with the non-OPLL group (Fig. 1a). Similarly, compared with the non-OPLL group, there was also increased expression of LC3 observed in the OPLL group (Fig. 1b). However, the expression level of ULK1 in the OPLL tissue was similar to that of the mean level of non-OPLL tissues (Fig. 1c).

### Correlation between rate of autophagy and osteogenic differentiation markers

Next, correlation was analyzed between the expression of autophagy-related genes (LC3 and Beclin1 mRNA) and osteogenic differentiation-related genes. Pearson correlation analysis demonstrated that the level of Beclin1 mRNA expression was positively correlated with the expression of ALP (*r* = 0.7821, *P* < 0.001) (Fig. 1d), COL 1 (*r* = 0.6078, *P* = 0.001) (Fig. 1e) and OCN (*r* = 0.8233, *P* < 0.001) (Fig. 1f). However, the expression of LC3 mRNA was not correlated with ALP (*r* = 0.1189, *P* = 0.6076) (Fig. 1g), COL 1 (*r* = − 0.07361, *P* = 0.7512) (Fig. 1h), and OCN (*r* = 0.11, *P* = 0.635) (Fig. 1i).

### Identification of ligament fibroblasts and growth characterization

**Fig. 2:**
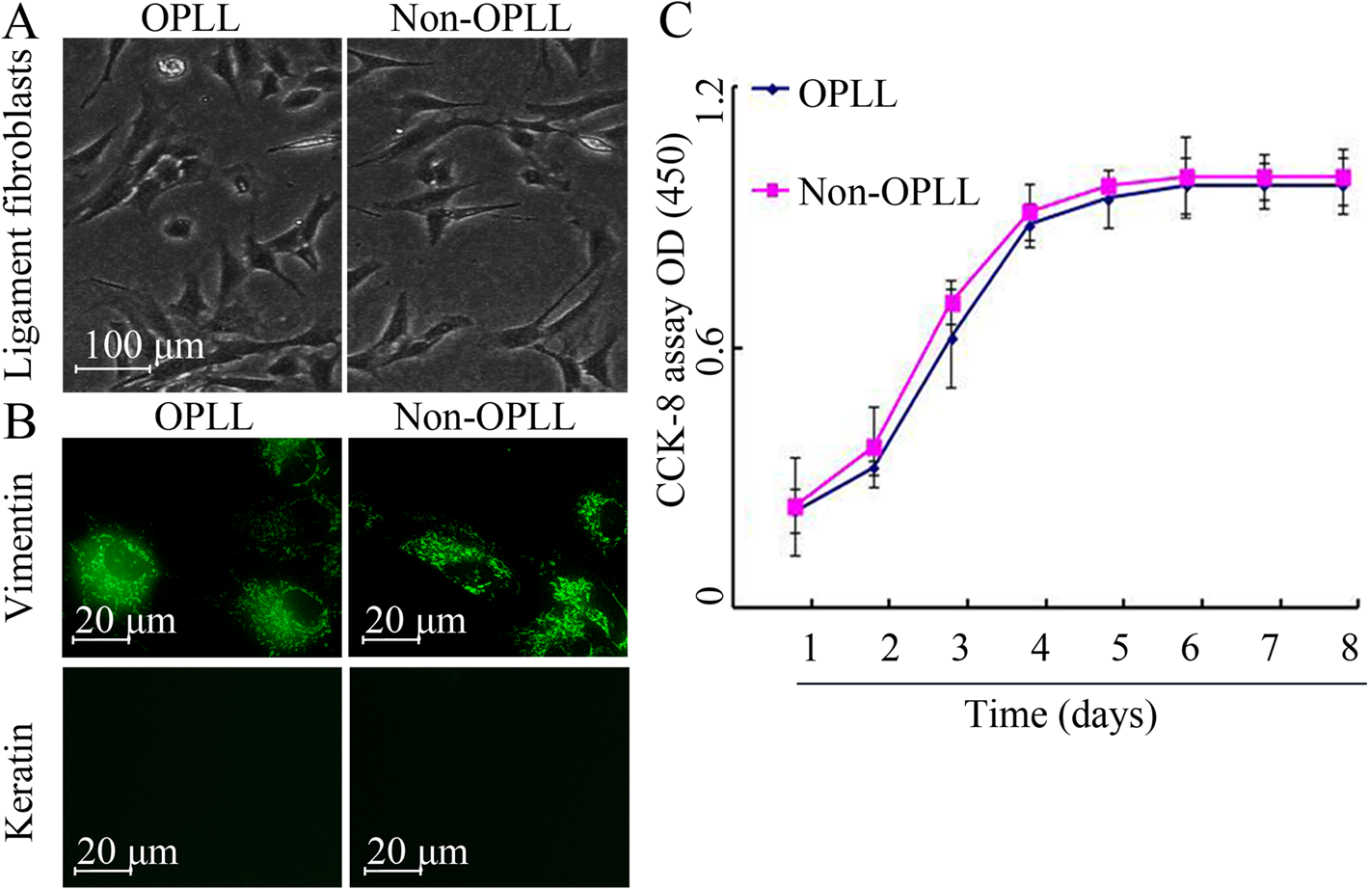
Identification of ligament fibroblasts and growth characterization. **a** Optical microscopy analysis of ligament fibroblasts derived from OPLL and non-OPLL patients (*n* = 12 for each group). The cells were multi-angled in shape and also had relatively long cell antennas. **b** Representative fluorescent images showing immunofluorescence assay for vimentin and keratin expression in ligament fibroblasts derived from OPLL and non-OPLL patients (*n* = 12 for each group). Scale bar, 20 μm. **c** CCK-8 assay measuring cell viability. Ligament fibroblasts derived from OPLL and non-OPLL patients were cultured in DMEM for 1, 2, 3, 4, 5, 6, 7, or 8 days (*n* = 12 for each group). ^*^P < 0.05 OPLL vs. non-OPLL. OPLL, ossification of the posterior longitudinal ligament

Next, ligament fibroblasts were isolated from tissue for further study (from OPLL and non-OPLL patients). After a 1-week tissue fragment culture, slender cells could be observed surrounding the isolated tissue fragments. They were multi-angled in shape and also had relatively long cell antennas. As shown in Fig. 2a, cells derived from the cervical posterior longitudinal ligaments of both OPLL and non-OPLL patients showed similar fibroblast-like morphological characters in size, nucleus, and shape (Fig. 2a). To further identify ligament fibroblasts, immunofluorescence staining was performed to evaluate the expression of vimentin and keratin. As expected, vimentin was generally expressed in the cytoplasm of the cells derived from both OPLL and non-OPLL ligament fibroblasts, and keratin was not detected in these ligament fibroblasts (Fig. 2b), which indicated that cells derived from patients with OPLL and non-OPLL patients showed similar fibroblast-like morphological characters in size, nucleus, and shape (Fig. 2c).

### Different expression of autophagy-specific gene makers in cells from OPLL and non-OPLL patients

**Fig. 3:**
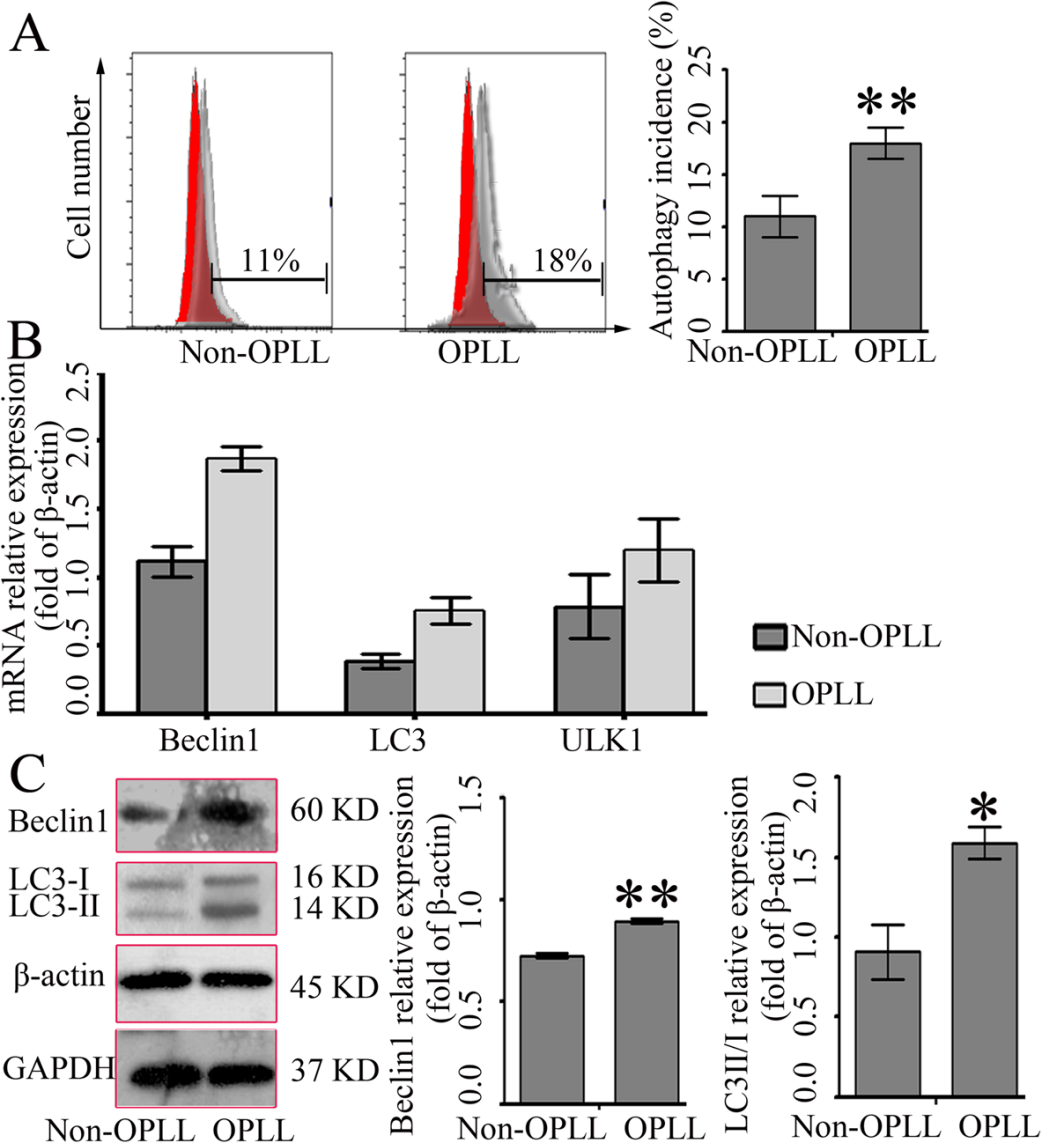
**a** Flow cytometry for autophagy incidence using monodansylcadaverine staining. The incidence of autophagy in OPLL-derived ligament fibroblasts was 1.6-fold of those in the non-OPLL-deprived ligament fibroblasts (*n* = 6 for each group). **b** RT-qPCR assay for Beclin1 and LC3 mRNA expressions. The expression levels of LC3 and Beclin1 mRNA increased in the OPLL-derived ligament fibroblasts compared with the non-OPLL-deprived ligament fibroblasts (*n* = 12 for each group). **c** Western blotting for LC3 and Beclin1 proteins. The expression levels of LC3 and Beclin1 proteins were significantly higher in the OPLL-derived ligament fibroblasts compared with the non-OPLL-deprived ligament fibroblasts (*n* = 12 for each group). ^*^P < 0.05, ^**^P < 0.01 OPLL vs. non-OPLL. OPLL, ossification of the posterior longitudinal ligament; RT-qPCR, reverse transcription-quantitative PCR; LC3, microtubule-associated protein 1 light chain 3

**Fig. 4:**
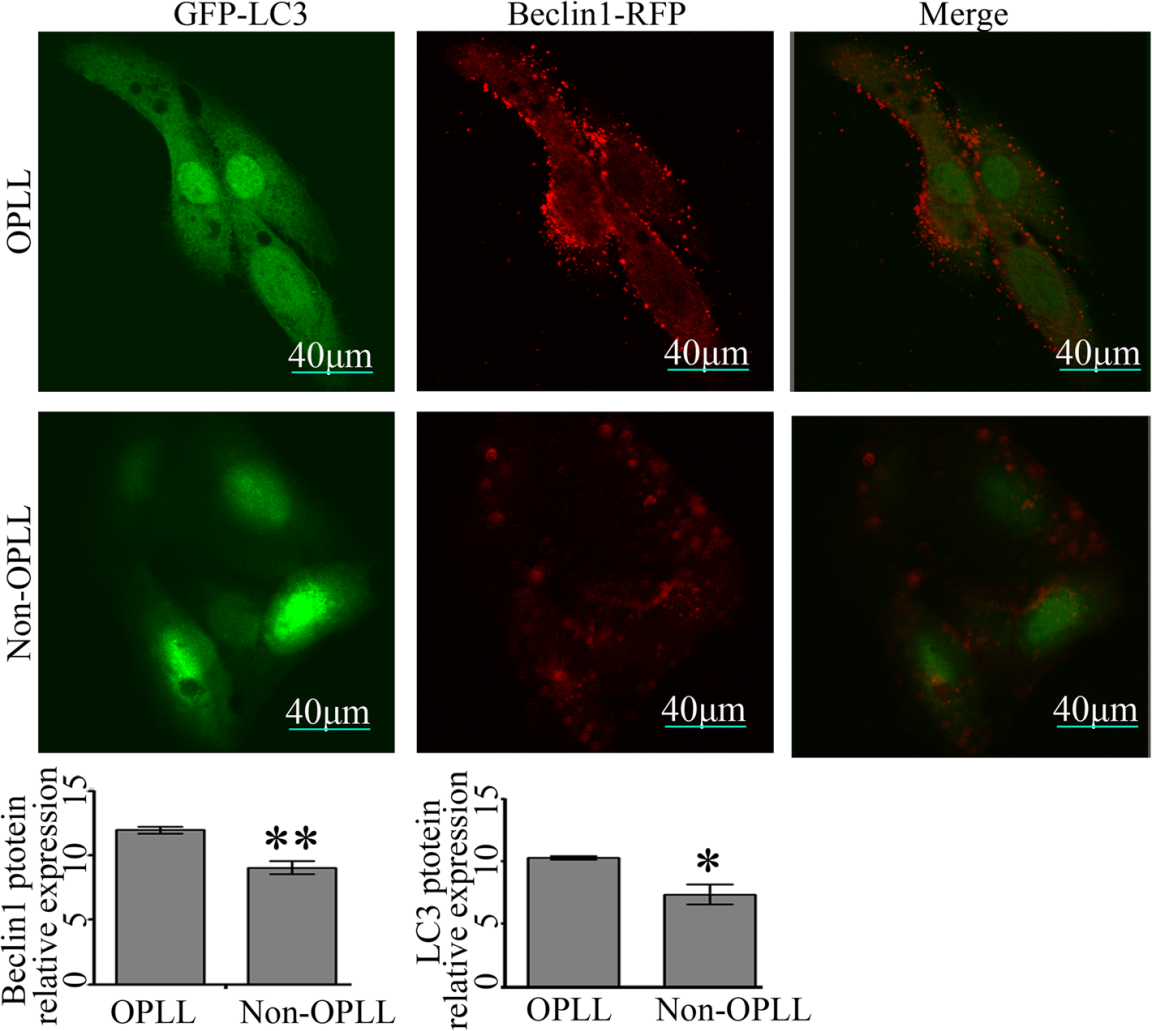
Representative fluorescence images showing immunofluorescence assay for LC3 protein (green), Beclin1 (red), and merged images under the two different conditions. Scale bar, 40 μm. An increase of Beclin1 and LC3 proteins were observed in the cytoplasm of the OPLL-derived ligament fibroblasts compared with the non-OPLL-deprived ligament fibroblasts (*n* = 12 for each group). ^*^P < 0.05, ^**^P < 0.01 OPLL vs. non-OPLL. OPLL, ossification of the posterior longitudinal ligament; LC3, microtubule-associated protein 1 light chain 3

In order to further confirm the results observed at the tissue level, flow cytometry assay, RT-qPCR, and western blotting were performed in ligament fibroblast cells. As shown in Fig. 3a, the flow cytometry assay indicated that the rates of autophagy in OPLL-derived ligament fibroblasts were 1.6-fold of those in the non-OPLL-deprived ligament fibroblasts. Consistent with the aforementioned results, RT-qPCR showed that the mRNA expression of LC3 and Beclin1 increased in ligament fibroblasts from patients with OPLL compared with fibroblasts from non-OPLL patients (Fig. 3b). Next, Beclin1 and LC3 protein expression was detected by western blotting, the expression levels of LC3 and Beclin1 proteins were significantly higher in ligament fibroblasts from patients with OPLL compared with the control (Fig. 3c). Consistently, fluorescence microscopy showed that Beclin1 and LC3 protein dots increased in the cytoplasm of ligament fibroblasts from patients with OPLL as compared with the group of ligament fibroblasts from non-OPLL patients (Fig. 4). Together, these data indicated that autophagy was upregulated in ligament fibroblasts from patients with OPLL.

### Role of Beclin1 in osteogenic differentiation of ligament fibroblasts

**Fig. 5:**
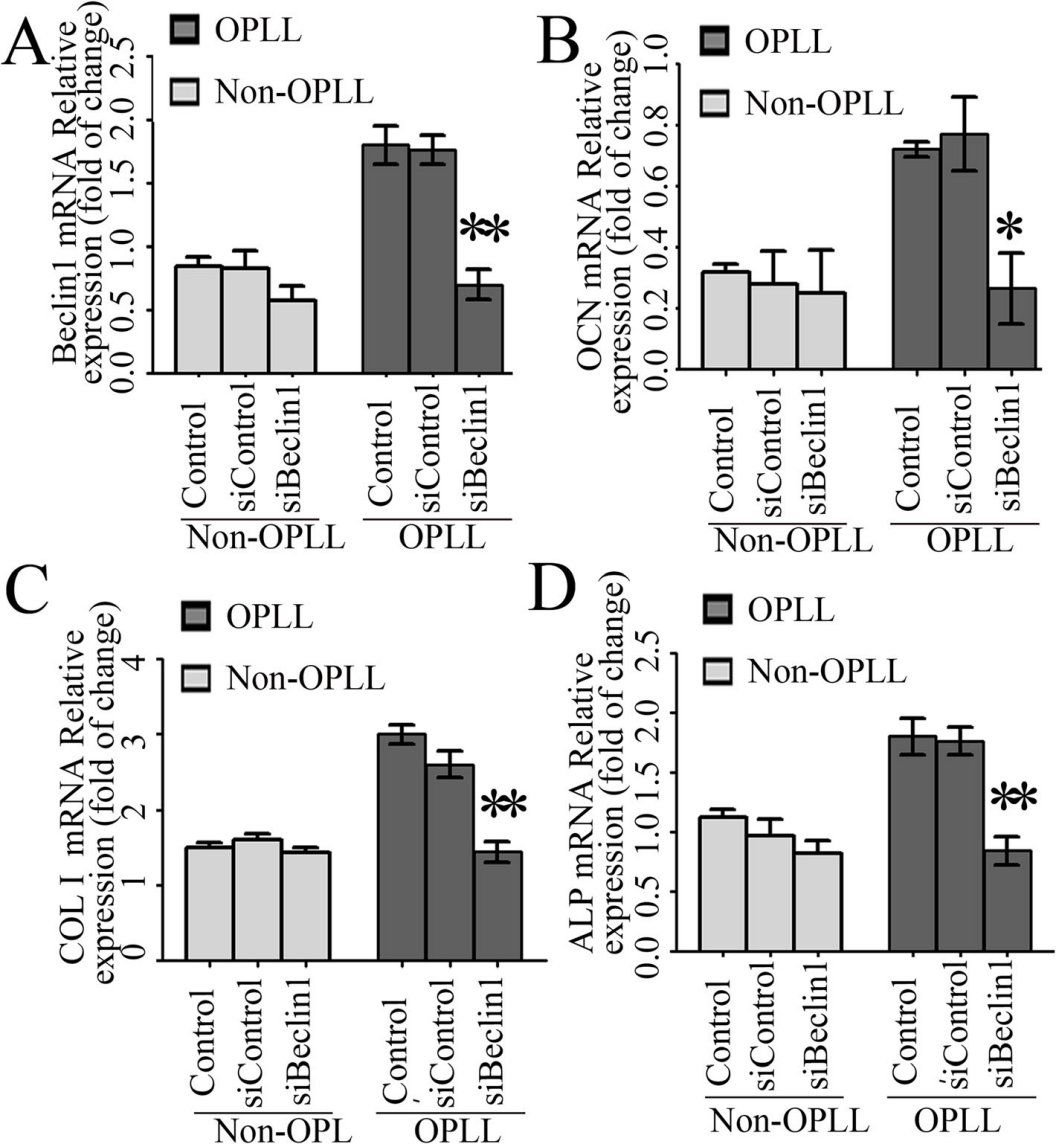
Role of Beclin1 in osteogenic differentiation of ligament fibroblasts. RT-qPCR assay for Beclin1 (**a**), OCN (**b**), COL 1 (**c**), and ALP (**d**) in OPLL and non-OPLL ligament fibroblasts transfected with siBeclin1 or siControl for 72 h. In OPLL ligament fibroblasts, after knockdown by siBeclin1, the expression of Beclin1, OCN, COL 1, and ALP genes were reduced by 63.2% (*P* < 0.01), 52% (*P* < 0.01), and 53.2% (*P* < 0.01), respectively. However, no significant difference was observed between the siBeclin1, siControl, and control groups in non-OPLL ligament fibroblasts (*n* = 12 for each group). OPLL, ossification of the posterior longitudinal ligament; RT-qPCR, reverse transcription-quantitative PCR; ALP, alkaline phosphatase, biomineralization associated; COL 1, collagen type 1; OCN, osteocalcin; si, small interfering

The aforementioned data indicated that Beclin1 was closely correlated with osteogenic differentiation markers and the expression of Beclin1 was significantly upregulated in the spinal ligament fibroblasts derived from patients with OPLL. Therefore, a specific gene interference technique was used, targeting Beclin1, to suppress the expression of Beclin1 in ligament fibroblasts. After 72 h of transfection in the ligament fibroblasts from patients with OPLL, it was found that Beclin1 expression decreased significantly by 61% (*P* < 0.0001) in the transfected cells, as measured by RT-qPCR (Fig. 5a). However, in the ligament fibroblasts from non-OPLL patients, there was no significant difference in Beclin1 expression among groups (*p* = 0.173) (Fig. 5a). The low efficiency in the knockdown of Beclin1 in the non-OPLL group could be because the basal autophagy level was low in the ligament fibroblasts from non-OPLL patients.

Subsequently, to clarify whether there are different roles of Beclin1 in osteogenic differentiation, mRNA expression of OCN, COL 1, and ALP was detected and then compared between the OPLL group and the non-OPLL group. Before transfection, the expressions levels of OCN (Fig. 5b), COL 1 (Fig. 5c), and ALP (Fig. 5d) were 2.71- (*P* < 0.001), 3.31- (*P* < 0.001), and 2.71 (*P* < 0.001)-fold of those in the non-OPLL cells, respectively. However, after transfection for 72 h, the expression of OCN, ALP, and COL 1 was reduced by 63.2% (*P* < 0.01), 52% (*P* < 0.01), and 53.2% (*P* < 0.01) in ligament fibroblasts from patients with OPLL compared with 3.1% (*P* > 0.01), 6.8% (*P* > 0.01), and 10.7% (*P* > 0.01) in the non-OPLL patients, respectively (Fig. 5b–d).

## Discussion

OPLL, which is characterized by ectopic bone formation in the spinal ligament, is a multi-factorial disease involving genetic, physical, and neurological disorders [1, 19]. The pathogenesis of OPLL is not completely understood. Current research suggests that OPLL is possibly associated with abnormal expression of connexin43 [20], PERK [19], and TGF-β [24]. However, these results were not consistent and further research is needed to elucidate the major genes that cause the susceptibility to OPLL.

In the present study, expression of ULK1, Beclin1, and LC3 were measured, which showed that Beclin1 and LC3 expression was significantly increased in OPLL tissues compared with non-OPLL tissues. Next, the association between the expression of Beclin1, LC3, and the osteogenic differentiation makers in 21 patients with OPLL were analyzed. As a result, it was found that Beclin1 mRNA was positively correlated with osteogenic differentiation makers. However, LC3 expression was not significantly correlated with osteogenic differentiation makers. A possible explanation for this is that LC3 mRNA level is not a very reliable measurement for changes in autophagy [25].

Next, ligament fibroblasts were isolated from tissues for further study (from OPLL and non-OPLL patients). Consistently, there was a higher level of Beclin1 and LC3 mRNA expression in the posterior longitudinal ligament fibroblasts derived from patients with OPLL compared with those derived from non-OPLL patients. Analysis of cell growth and morphology demonstrated that no difference was observed in cells from OPLL and non-OPLL patients in vitro. Although, it was not consistent with a previous study that reported that cells from patients with OPLL were larger in size and nucleus [19]. The main reason for this could be that cells were derived from different populations. Previous data suggests that autophagy is essential for osteogenic differentiation [18]. To confirm the results at the tissue level, the mRNA and protein expression of autophagy-related genes were further analyzed in ligament fibroblasts derived from OPLL and non-OPLL patients. Consistently, the present results indicated that the level of autophagy in ligament fibroblasts from patients with OPLL were higher compared with those derived from non-OPLL patients. These data suggested that autophagy was increased in ligament fibroblasts, in particular in ligament fibroblasts from patients with OPLL.

**Fig. 6:**
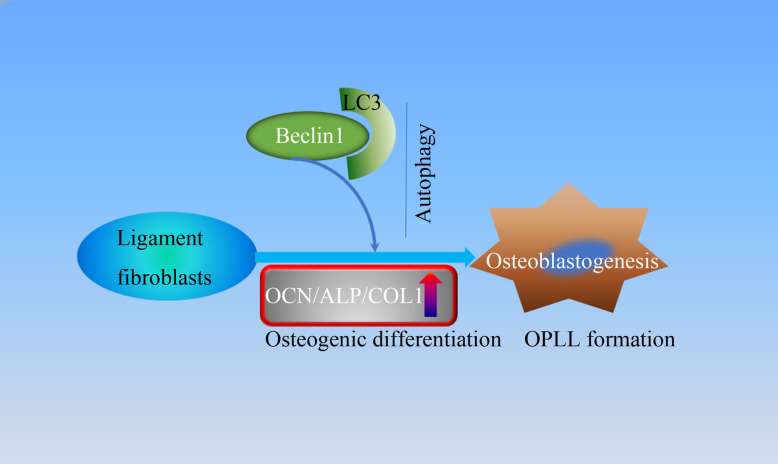
A hypothetic diagram for pathogenesis of OPLL. Aberrant activation of Beclin1-autophagy signaling plays an important role in development of OPLL. Beclin1-autophagy is upregulated in OPLL-derived ligament fibroblast and transcriptions of OCN, ALP, and COL 1 are increased, which promotes the differentiation of ligament fibroblast and ultimately causes OPLL formation. OPLL, ossification of the posterior longitudinal ligament; ALP, alkaline phosphatase, biomineralization associated; COL 1, collagen type 1; OCN, osteocalcin

Fibroblast cells can differentiate into osteoblasts or osteocytes [26]. Therefore, the expression levels of three marker genes related to osteogenic differentiation, OCN, ALP, and COL 1, were assessed. It was found that the mRNA expression of osteogenic differentiation markers OCN, COL 1, and ALP were significantly upregulated in the OPLL group [27] compared with the control. Furthermore, analysis of gene expression demonstrated a higher mRNA and protein expression of Beclin1 and LC3II in the fibroblasts from patients with OPLL. Notably, Beclin1 expression was correlated with the expression of osteogenic differentiation markers OCN, ALP, and COL 1 in patients with OPLL. Consistently, a recent study reported that Beclin1 plays an important role in the differentiation of osteoclasts and chondrocytes [9]. To confirm the role of Beclin1 in fibroblasts, siRNA targeting Beclin1 was designed and used to downregulate the expression of Beclin1. As expected, a significant decrease in the expression of Beclin1 was observed in the fibroblasts from patients with OPLL, while a slight reduction of Beclin1 expression, which was not significantly different, was found in the non-OPLL group. These results are consistent with previous researches that the effect of siRNA is dependent on its relative expression level [19, 20]. Similarly, in ligament fibroblasts derived from patients with OPLL, the expression levels of OCN, ALP, and COL 1 were significantly suppressed by knocking down Beclin1 compared with the control. However, no significant difference was observed in fibroblasts from non-OPLL patients. The present results suggested that Beclin1-autophagy was involved in the process of osteogenic differentiation in OPLL (Fig. 6). Consistently, in our previous study, autophagy was confirmed in murine ligament fibroblasts and suppression of autophagy with either pharmacologic inhibitors or Beclin1 knocking down weakened the mineralization capacity of ligament fibroblasts [28].

Although our research is the first to provide data demonstrating that autophagy plays a role in the development of OPLL. However, OPLL is a multi-factorial disease in which complex genetic and environmental factors interact. Obviously, the cell biological data in vitro provides very limited evidence. Furthermore, control group was taken from the patients having cervical spinal fracture. However, some of them may develop OPLL thereafter due to aging, diabetes mellitus, obesity, and susceptibility genetic loci. Thus, further research using strict inclusion and exclusion criteria is indispensable to confirm the findings of this study.

To conclude, Beclin1-mediated autophagy was involved in the osteogenic differentiation of ligament fibroblasts and promoted the development of OPLL.

## Conclusions

Our findings suggested that autophagy is upregulated in spinal ligament fibroblasts derived from OPLL patients and Beclin1-mediated autophagy was involved in the osteogenic differentiation of ligament fibroblasts and promoted the development of OPLL.

## Data Availability

All data generated or analyzed during this study are included in this published article.

## Abbreviations

OPLL: Ossification of the posterior longitudinal ligament
RT-qPCR: Reverse transcription-quantitative polymerase chain reaction
OCN: Osteocalcin
ALP: Alkaline phosphatase
COL 1: Collagen type 1
ULK1: Unc-51 like autophagy activating kinase 1
LC3: Microtubule-associated protein 1 light chain 3
siRNA: Small interfering RNA
MDC: Monodansylcadaverine
CT: Computed tomography
MRI: Magnetic resonance imaging;
